# Sensorimotor Representation of Speech Perception. Cross-Decoding of Place of Articulation Features during Selective Attention to Syllables in 7T fMRI

**DOI:** 10.1523/ENEURO.0252-17.2018

**Published:** 2018-04-02

**Authors:** Mario E. Archila-Meléndez, Giancarlo Valente, Joao M. Correia, Rob P. W. Rouhl, Vivianne H. van Kranen-Mastenbroek, Bernadette M. Jansma

**Affiliations:** 1 Department of Cognitive Neuroscience, Faculty of Psychology and Neuroscience, Maastricht University, Maastricht 6229 EV, The Netherlands; 2 Maastricht Brain Imaging Center (M-BIC), Maastricht University, Maastricht 6229 EV, The Netherlands; 3 Department of Neurology, Maastricht University Medical Center+, Maastricht 6202 AZ, The Netherlands; 4 School for Mental Health and Neuroscience (MHeNS), Maastricht University, Maastricht 6200 MD, The Netherlands; 5 Department of Clinical Neurophysiology, Maastricht University Medical Center+, Maastricht 6229 HX, The Netherlands; 6 Center for Integrative Neuroscience (CIN), Maastricht University, Maastricht 6200 MD, The Netherlands; 7 Academic Center for Epileptology Kempenhaeghe/Maastricht University Medical Center+, Maastricht 6202 AZ, The Netherlands

**Keywords:** 7T fMRI, MVPA-based cross-decoding, place of articulation, selective attention, sensorimotor, speech perception

## Abstract

Sensorimotor integration, the translation between acoustic signals and motoric programs, may constitute a crucial mechanism for speech. During speech perception, the acoustic-motoric translations include the recruitment of cortical areas for the representation of speech articulatory features, such as place of articulation. Selective attention can shape the processing and performance of speech perception tasks. Whether and where sensorimotor integration takes place during attentive speech perception remains to be explored. Here, we investigate articulatory feature representations of spoken consonant-vowel (CV) syllables during two distinct tasks. Fourteen healthy humans attended to either the vowel or the consonant within a syllable in separate delayed-match-to-sample tasks. Single-trial fMRI blood oxygenation level-dependent (BOLD) responses from perception periods were analyzed using multivariate pattern classification and a searchlight approach to reveal neural activation patterns sensitive to the processing of place of articulation (i.e., bilabial/labiodental vs. alveolar). To isolate place of articulation representation from acoustic covariation, we applied a cross-decoding (generalization) procedure across distinct features of manner of articulation (i.e., stop, fricative, and nasal). We found evidence for the representation of place of articulation across tasks and in both tasks separately: for attention to vowels, generalization maps included bilateral clusters of superior and posterior temporal, insular, and frontal regions; for attention to consonants, generalization maps encompassed clusters in temporoparietal, insular, and frontal regions within the right hemisphere only. Our results specify the cortical representation of place of articulation features generalized across manner of articulation during attentive syllable perception, thus supporting sensorimotor integration during attentive speech perception and demonstrating the value of generalization.

## Significance Statement

Speech is supported by sensorimotor integration, a bidirectional translation of its auditory and motoric signals. Whether our brain represents speech as articulatory features during selective attention has not yet been well specified. We focused on the representation of articulatory information of speech during attentive speech perception. For the first time, we applied generalization in classification analysis to counteract the differences in acoustic properties that accompany articulatory information. Participants attended to either the vowels or consonants of syllables, while undergoing fMRI. Our results show that articulatory information is represented in widespread cortical areas during selective attention to the different syllable components, supporting sensorimotor integration during attentive speech perception.

## Introduction

Speech is supported by sensorimotor integration, a (bidirectional) translation of its auditory and motoric signals. These translations, which occur during speech perception (Hickok and Poeppel, 2007; Evans et al., 2016; Schomers and Pulvermüller, 2016), can result in the cortical representation of articulatory features of speech, such as place of articulation, manner of articulation, and voicing. Particularly, the cortical representation of place of articulation features has been reported in dorsal speech regions, including motor and premotor areas (Pulvermuller et al., 2006), and somatosensory and supramarginal regions under passive listening (Correia et al., 2015). However, differential task requirements can modulate cortical representations of articulatory features. For example, variation of somatotopic activations in motor areas were found during passive sound perception involving different articulators (Pulvermuller et al., 2006). Other researchers have reported differential patterns in superior temporal but not in the motor cortex during an incidental task with phonemes (Arsenault and Buchsbaum, 2015). Several reasons have been discussed to account for this variability; among them, differences in task demands across studies (e.g., type of task, number of items, but also passive versus active tasks, i.e., selective attention) as well as third factors such a covariation of manner of articulation during place processing.

Although the underlying neural mechanisms of speech perception and attention remain elusive, they are explained in terms of network dynamics (Friederici and Singer, 2015) and, in particular, of theta-γ amplitude or phase coupling (Giraud and Poeppel, 2012; Hyafil et al., 2015) of neural activity. For example, attention can implement phase resetting and entrainment of neuronal oscillations to a relevant stimulus stream (Lakatos et al., 2008) and can rapidly change the spectrotemporal receptive field to enhance task-relevant stimulus properties (Fritz et al., 2003). Importantly, selective attention has been shown to generate spatial coupling patterns between prefrontal and feature-specific cortical areas (Baldauf and Desimone, 2014).

Ultra high-field 7 Tesla fMRI allows investigating the living human brain with unprecedented high spatial resolution (Yacoub et al., 2001), signal-to-noise ratio (Vaughan et al., 2001), and specificity (Polimeni et al., 2010). Beyond measurement improvements, multivariate pattern analysis (MVPA) further increases the sensitivity of experimental contrasts by exploiting concurrent spatial patterns of fMRI responses (Norman et al., 2006). Crucially, MVPA has allowed unraveling information representation of abstract speech features (Correia et al., 2015; Evans and Davis, 2015).

Here, we aim to minimize the effect of covariation in manner of articulation during place of articualtion processing by generalization across manner. We study the cortical representation of place of articulation features of syllables using 7T fMRI and MVPA-based cross-decoding (i.e., generalization). Specifically, we exploited the acoustic variation imposed by two manners of articulation (e.g., stop and fricative) to identify patterns discriminative of place of articulation (i.e., bilabial/labiodental versus alveolar) and tested these patterns in a third (unseen) manner of articulation (e.g., nasal). This procedure capitalized on the acoustic variation imposed by different manner of articulation features to extract specific patterns for place of articulation features (Ladefoged and Johnson, 2010).

This generalization was applied to two different tasks with identical auditory stimuli (i.e., attention to consonants and attention to vowels) to investigate the neural representation of place of articulation features during selective attention to speech and the possible effects of attention on the neural representation. Previous studies have shown representation of place of articulation features in sensorimotor regions (Correia et al., 2015) and a modulatory role of attention in sound and phonetic representations of speech (Bonte et al., 2014; Downer et al., 2015). Based on these studies, we used decoding of place of articulation features as a metric of sensorimotor processes during speech perception. We then investigated whether decoding was possible on either task and, if so, whether sensorimotor integration is modulated by the selective attention to different syllable components (i.e., vowel or consonants). The idea behind choosing vowels and consonants tasks was that place of articulation is more representative for consonant identification, and less for vowel identification in consonant-vowel (CV) structures. This orthogonal stimuli-task arrangement allowed assessing the effect of attention on the place of articulation representation without explicit task focus to place.

### Participants

Fourteen native Dutch speakers (mean ± SD age, 27.2 ± 5.1 years; nine females; two left-handed) underwent blood oxygenation level-dependent (BOLD) signal fMRI scanning while performing two delayed-match-to-sample tasks. Participants had no history of neurologic or systemic diseases and reported normal hearing abilities. All participants received monetary compensation for their participation and signed written informed consent. The Ethical Committee of the Faculty of Psychology and Neuroscience at the University of Maastricht, Maastricht, The Netherlands approved the study.

### Stimuli

**Figure 1.**
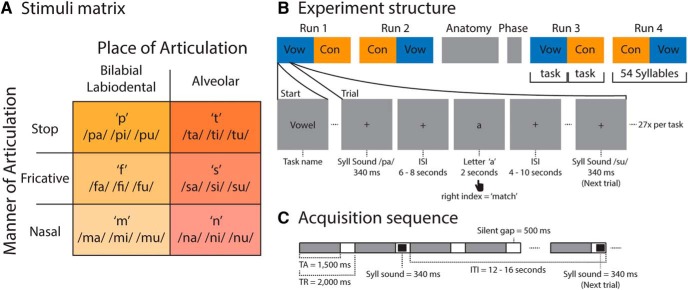
Stimuli and experimental paradigm. ***A***, Spoken stimuli matrix and articulatory properties. The 18 syllables were selected according to the place of articulation (i.e., bilabial/labiodental and alveolar) and manner of articulation (i.e., stop, fricative, and nasal). ***B***, Experimental procedure and task description. Example of a typical match-to-sample trial during attend to vowels task. Subjects received instructions per block, in which they attended to consonants or vowels, respectively, and conducted a match-to-sample decision within slow-event related trials. Each block started with the visual presentation of a task cue (i.e., attention target vowel or consonant), indicating which task to perform in the next 27 trials. Each trial started with a fixation period in which a fixation cross was presented at the center of screen, together with a syllable sound (340 ms). After a jittered ISI (jitter: 6–8 s) a visual cue (i.e., a written letter, vowel, or consonant) was presented for 2 s, followed by the immediate subject’s response by pressing a button either with the right index finger (for match trials) or middle index finger (for mismatch trials). The response was followed by a jittered ISI (4–10 s) to complete the jittered ITI period (12–16 s) before the next trial started. ***C***, Schematic representation of the fMRI acquisition sequence and its relationship to the syllable sounds presented to the participants. Vow, attentd to vowels task; Con, attend to consonants task; Syll, syllable; TA, time of acquisition; TR, time for repetition; Phase, opposite phase encoding volumes acquired for distortion correction.

Stimuli were 18 CV syllables recorded by three female native Dutch speakers, generating 54 unique tokens. The syllables were the result of the CV combinations of six consonants (p, t, f, s, m, n) and three vowels (a, i, u). This subset of consonants was selected because it allowed us to build a balanced stimuli matrix that covers two features of place of articulation (i.e., bilabial/labiodental and alveolar) and three features of manner of articulation (i.e., stop, fricative, and nasal). This arrangement was needed to perform MVPA-based cross-decoding analysis and to keep the necessary number of trials for single-trial classification (see below, Multivariate analysis). Syllable recordings were selected from a subset of recordings previously used in our laboratory (Correia et al., 2015). Briefly, the consonants composing the syllables for every articulatory feature were bilabial/labiodental (p, f, m) and alveolar (t, s, n) for place of articulation; and stop (p, t), fricative (f, s), and nasal (m, n) for manner of articulation (Fig. 1*A*). The three different vowels and three different speakers introduced acoustic variability useful for classification. All stimuli were recorded in a soundproof chamber at a sampling rate of 44.1 kHz and digital-to-analog converted with 16-bit resolution. Stimuli were presented in the MRI scanner via MR-compatible earphones with a linear frequency transfer of up to 8 kHz (model S14, Sensimetrics Corporation). Before starting the experiment, the volume was adapted to each subject’s audible and comfortable perceptual level.

### Experimental design

Participants performed two delayed-match-to-sample tasks in four runs of 54 trials each (i.e., each syllable token was presented once per run) during fMRI acquisition. In cognitive terms, this task allowed paying attention to a certain aspect of the acoustic stimuli, from now on referred to as “attend to vowels” and “attend to consonants.” The task also involved remembering and matching the attended vowel or consonant later on with a visually presented token (i.e., a written vowel or consonant) of the same category (with 50% match/mismatch response proportions). Each run was divided into two blocks of 27 trials, one for each task, and the tasks where counterbalanced across runs and participants. During the attend to vowels task, participants heard a syllable and received, after 6–8 s, a written vowel as “match cue.” Participants were instructed to match the vowel of an auditorily presented CV syllable with a written vowel cue as fast and accurately as possible by pressing a button with the right index (“match”) or middle (“mismatch”) finger. During the attend to consonants task, participants heard the same syllables (of the attend to vowels task) and received a written consonant as cue that should be matched with the auditory syllable (Fig. 1*B*). Importantly, the features of interest (i.e., place of articulation) were never part of an explicit task to avoid confounds in attentional demands. The delay-match-to-sample task is widely used to precisely control the subject’s attention. Thus, data were analyzed only for the first part of each trial before the cue onset to avoid that relevant signal was contaminated with matching and response related processing period signals.

The task was clearly explained to each subject outside the scanner, and they received at the beginning of each block an introduction display indicating the syllable component they should attend to (i.e., vowels or consonants) at the beginning of each task. A trial consisted of (1) a speech stimulus sound presentation (i.e., a CV syllable token of 340-ms duration) followed, after an interstimulus interval (ISI) of 6-8 s; by (2) a written cue (i.e., a consonant or vowel letter matching or not the sound token depending on the specific block and trial of 2 s duration, written in Times New Roman, font size 30, black color); and then followed by (3) the subject’s match/mismatch immediate response. The sound syllables and the visual cue (i.e., a written vowel or consonant) matched in 50% of the trials, and match/mismatch trials were balanced across attention conditions and randomized per subject. Mismatch cues were always of the same category (i.e., vowels in the attend to vowels task and consonants in the attend to consonants task). In total, each run lasted 15 min and the behavioral responses were collected through an MR-compatible button box (Current Designs, eight-button response device, HHSC-2x4-C).

We used fast sparse image acquisition to have a 500-ms silent gap to present each syllable sound (Di Salle et al., 2003). The fMRI acquisition was set up as a slow event-related design. The intertrial interval (ITI) between consecutive auditory stimuli was relatively long (i.e., 14 s on average; range: 12–16 s) to allow independent BOLD signal estimation per trial (Fig. 1*C*). The interval used to estimate the fMRI response to the spoken syllable perception per trial was restricted to the first 6 s following the sound presentation to avoid contamination from the processing of the visual cue, matching, or button press. Moreover, as the acoustic, phonetic, and phonological features of the presented syllables were identical across trials and attention conditions, the effects on the cortical representations pertain to differences in attention. All stimuli, event identities, and timings were presented and logged using Presentation from Neurobehavioral Systems (www.neurobs.com; RRID: SCR_002521).

### Functional MRI acquisition

Functional and anatomic volumes were acquired on a whole-body Siemens Magnetom 7 Tesla scanner (Siemens) and a 32-channel head-coil (Nova Medical Inc.) at the Maastricht University Medical Center Imaging Center. For all functional runs, we acquired whole brain high-resolution accelerated multiband gradient echo (T2*-weighted) echo-planar imaging (EPI; Moeller et al., 2010; Setsompop et al., 2012) data [echo time (TE) = 21 ms; repetition time (TR) = 2000 ms; time of acquisition (TA) = 1500; delay in TR (silent gap) = 500 ms; multi-band factor = 3; generalized auto-calibrating partially parallel acquisitions (GRAPPAs) g-factor = 2; flip angle = 72°; field of view, FOV = 198 mm; voxel size = 1.5 × 1.5 × 1.5 mm^3^; number of slices = 72, without gap between slices] for each participant. To correct for EPI geometric distortions, 10 volumes with opposite phase encoding directions (i.e., posterior to anterior and anterior to posterior) were additionally acquired using the same acquisition parameters as in the functional runs. After the first 2 functional runs, we acquired a tridimensional T1-weighted magnetization prepared rapid acquisition gradient echo (3D-MP2RAGE; Marques et al., 2010) volume (240 sagittal slices; voxel size = 0.65 × 0.65 × 0.65 mm^3^; first inversion time TI1 = 900 ms; second inversion time TI2 = 2750 ms; TE = 2.51 ms; TR = 5000 ms; first nominal flip angle = 5°; second nominal flip angle = 3°) per participant.

### Functional MRI preprocessing

Anatomic and functional data were analyzed using BrainVoyager QX (version 2.8.4; Brain Innovation; RRID: SCR_013057), and custom code written in MATLAB (R2014a version 8.3.0.532; The Mathworks Inc.; RRID: SCR_001622). Anatomic images were interpolated to a nominal voxel size of 1.5 × 1.5 × 1.5 mm^3^ matching the functional images’ resolution. The functional images were corrected for motion artifacts using the 3D rigid body motion correction algorithm implemented in BrainVoyager QX and all functional runs were aligned to the first volume of the second functional run. We corrected the EPI distortions using the *topup* tool implemented in FSL (RRID: SCR_002823; Smith et al., 2004). The reversed phase encoding images, acquired after the anatomic images, were used to estimate the susceptibility-induced off-resonance field and, then, to correct the distortions in the remaining functional runs. After this correction, functional data were high-pass filtered using a general linear model (GLM) Fourier basis set of eleven cycles sine/cosine, including a linear trend removal. Functional volumes per run were co-registered and aligned to the anatomic scan using rigid body transformations (i.e., six parameters: three translations and three rotations). Finally, functional images were normalized by transformation into Talairach space (Jean and Tournoux, 1988).

#### Anatomic segmentation and cortex-based alignment (CBA)

Intensity inhomogeneities in T1-weighted images were first corrected using Statistical Parametric Mapping 12 (SPM12; RRID: SCR_007037; Ashburner and Friston, 2005) software. The resulting images were used to perform volumetric segmentation with the FreeSurfer analysis software (version 5.3.0, http://surfer.nmr.mgh.harvard.edu/; RRID: SCR_001847; Dale et al., 1999). Briefly, this processing includes motion correction, removal of non-brain tissue using a hybrid watershed/surface deformation procedure, automated Talairach transformation, intensity normalization, tessellation of the gray/white matter boundary, automated topology correction, and surface deformation following intensity gradients to optimally place the gray/white and gray/cerebrospinal fluid borders at the location where the greatest shift in intensity defines the transition to the other tissue class. Quality control was performed by visually inspecting each subject’s brain after the process was finished. Remaining errors were manually corrected using ITK-SNAP software (version 3.4.0, www.itksnap.org; RRID: SCR_002010; Yushkevich et al., 2006). The resulting binary maps were then used to reconstruct individual 3D meshes of the cortical surfaces and aligned using a moving target-group average approach based on curvature information (i.e., CBA) to obtain an anatomically aligned group-averaged 3D surface representation of all the subjects (Goebel et al., 2006; Frost and Goebel, 2012). Functional data were analyzed (see below, Multivariate analysis) in the volume space and then projected to the average surface, to perform group statistics and visualization in the aligned CBA space.

### Univariate analysis

Single subject GLM analysis was performed on fMRI signal time courses normalized with percentage transform in volume space. Next, they were mapped onto surface space by sampling the values located between 1 mm below the gray/white matter boundary and up to 3 mm into the gray matter toward the pial surface using trilinear interpolation and averaging. This sampling resulted in a single value per vertex in the subject’s cortex mesh, and then the values were aligned to the cortical group surface mesh using CBA. Random-effects GLM analysis was performed on these individual time course data. The conditions were collapsed across the speaker and vowel dimensions, thus yielding 12 predictors, six predictors for each consonant × two tasks. Each predictor was convolved with a canonical double gamma hemodynamic response function (HRF). Functional maps (i.e., average β values) were calculated to assess sound-evoked fMRI responses during attend to vowels and attend to consonants tasks (i.e., all sounds attend to vowels task > baseline; all sounds attend to consonants task > baseline). Task differences were analyzed for attend to consonants and attend to vowels task activity (i.e., attend to consonants task > attend to vowels task). Univariate stimulus effects were analyzed for each place of articulation feature independently (e.g., p_Con + f_Con + m_Con + p_Vow + f_Vow + m_Vow > t_Con + s_Con + n_Con + t_Vow + s_Vow + n_Vow). All functional contrast maps were corrected for multiple comparisons by applying a permutation-based cluster-size threshold with an initial cluster forming threshold of *p* = 0.05. The cluster-size threshold was based on the distribution of maximum cluster sizes obtained in the 2000 permutations, only considering clusters whose size was larger than the 95% quantile.

### Multivariate analysis

To investigate the cortical representations of the different articulatory features, we used MVPA in combination with a moving volumetric searchlight approach (Kriegeskorte et al., 2006). The purpose of the multivariate analysis was to decode articulatory features for each syllable independent from their specific phonetic signatures or acoustical properties. Therefore, we used a classification approach based on cross-decoding, generalizing place of articulation features across different dimensions of manner of articulation. Specifically, we trained a classifier to discriminate bilabial/labiodental versus alveolar places of articulation using syllables exhibiting two manners of articulation dimensions (e.g., stop and fricative) and tested whether this training was transferable to syllables exhibiting a third (unseen) manner dimension (e.g., nasal). MVPA generalization analysis was performed within each subject using all three combinations of generalization (i.e., leaving one manner of articulation out for testing at each split). The obtained averaged accuracies were submitted to a group analysis using random permutations for significance and nonparametric permutation-based cluster-size thresholding for multiple comparisons correction (see below, Multivariate statistical analysis, group statistics).

### Within-subject decoding

Before classification, BOLD time courses were selected by extracting the responses in each trial and each cortical voxel. The BOLD responses were estimated by fitting a standard HRF using multiple linear regression, taking the first three samples per trial after sound onset (i.e., corresponding to 6000 ms). The regression coefficients resulting from the fitting of single trial data in the cortical mask were used to build an fMRI feature space (i.e., number of trials by number of cortical voxels), which was then used in the multivariate decoding. During the multivariate decoding, we kept trials belonging to each task separate.

We limited our analysis to gray matter voxels using a subject-specific cortical ribbon mask based on intensity values in the T1-weighted images (i.e., cortical ribbon segmentation). We constrained our analysis to the cortical ribbon, as we were mainly interested in cortical processing. Ribbons allowed us to exclude volumes containing white matter and subcortical voxels from the analysis, which increased the number of dimensions. For the searchlight analysis, a sphere with radius of 5.5 voxels (i.e., 8.25 mm) was moved through the cortical ribbon, which defined a feature space of 65,000 voxels on average. In each searchlight, we performed generalization of place of articulation across manner of articulation using linear discriminant analysis (LDA) with diagonal covariance matrix. Given the large number of searchlights, we employed a fast Matlab implementation described in Ontivero-Ortega et al. (2017).

We also investigated the cortical representation of place of articulation independent of the task effect. We did so by performing classification and generalization across manner of articulation in the pooled data from the two tasks (i.e., attend to vowels and attend to consonants). By doing this, we use twice the amount of data as in the previous analyses and can expect more robust and reliable findings due to the power increase in single subject analyses. To avoid any task bias, we controlled for the number of trials belonging to each task in each cross-validation split maintaining a balanced contribution of each task during the training and testing procedures. The decoding was performed as described above.

### Multivariate statistical analysis, group statistics

In each subject, classification accuracies were obtained, separately for each task (i.e., attention to consonant and attention to vowel), for each searchlight centered on voxels belonging to the subject’s specific cortical ribbon. To perform a group analysis, these accuracies were projected onto each subject’s reconstructed cortical surface and then mapped back onto the group average cortical sheet using CBA. After the realignment, we considered in the group analysis only the vertices common to all subjects. To assess uncorrected significance of the classifications at the group level, we considered a non-parametric permutation test at each searchlight location independently. For each vertex, we took all the subject accuracies and the associated mean, and, using a resampling approach, we estimated the probability that such mean or a higher value could occur if the data were obtained under the null hypothesis (H0) that the decoding population mean is at chance level (i.e., 50%). This probability is the *p* value associated with the observed group mean. The resampling strategy was based on the fact that, under H0, the likelihood of the observed accuracies is symmetric around chance (i.e., 55% and 65% are equally likely if the population mean is 50%), and we can, therefore, build many datasets by switching some of the subjects’ accuracies around chance (Good, 2005, their section 3.2.1). We ran 2000 random permutations (Monte Carlo permutations) and for each searchlight we determined the *p* value as the ratio between how many times the mean of a population resample equaled or exceeded the observed mean and the total number of permutations (adding 1 to both numerator and denominator to avoid 0 *p* values, hence the lowest *p* value was 1/2001).

Finally, to correct for multiple comparisons, we employed a permutation-based cluster-size thresholding. We considered an initial Cluster Forming Threshold equal to 0.05 and, then, for each permutation, we tagged as significant those vertices whose observed mean was higher than the 1- quantile (obtained with the resampling procedure described above). For each of these “false positive” maps, we determined the maximum extent of clusters (on the surface) and built a distribution of cluster sizes across the 2000 permutations. Clusters of significant vertices, or equal to 0.05, in the observed data that were larger than the 95% quantile of the obtained distribution were deemed significant and corrected for multiple comparisons. It is worth mentioning that this procedure is based on a permutation strategy that does not suffer from inflation of false positive rate, as recently shown in Eklund et al. (2016).

## Results

### Behavioral results in fMRI experiment

To test whether there were any significant difference in reaction times, a factorial ANOVA was conducted, with task (attention to vowels and attention to consonants), place of articulation (bilabial/labiodental and alveolar), manner of articulation (stop, fricative, and nasal), speaker (speaker 1, speaker 2, and speaker 3), vowel (a, i, and u), and subject (subject 1-14, treated as random factor) as main factors on correct trials only. There was no effect across conditions in behavior. Reaction times for the vowel (mean = 843 ms, SD = 402) and the consonant task (mean = 820 ms, SD = 331) did not significantly differ (*F*_(1,2541)_ = 1.26, *p* = 0.261). There were no significant main effects or interactions (all *p* > 0.163).

### Functional MRI univariate results during the attend to consonants and attend to vowels tasks

**Figure 2.**
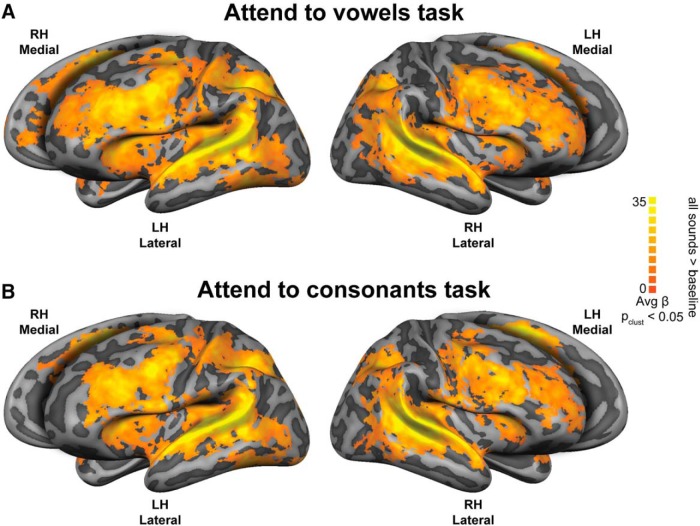
Brain areas that show speech sound processing during attend to vowels and attend to consonants tasks. Functional maps depicting the overall pattern of sound-evoked cortical responses during performance of (***A***) attend to vowels (i.e., all sounds attend to vowels > baseline) and (***B***) attend to consonants task (i.e., all sounds attend to consonants > baseline); *p* value at cluster threshold (*p*-clust) < 0.05. Maps are visualized on inflated and aligned group-averaged representations of the left (LH) and right (RH) hemispheres of the fourteen subjects (light gray, gyri; dark gray, sulci). Color scale indicates average β values.

During both the attend to vowels and attend to consonants tasks, the stimuli evoked significant BOLD responses in an extensive area of the superior temporal cortex, encompassing early auditory areas (i.e., Heschl’s gyrus (HG) and Heshl’s sulcus), the planum temporale, the superior temporal sulcus (STS) and superior temporal gyrus (STG), and the posterior part of the middle temporal gyrus (MTG). Additionally, the insula, supramarginal gyrus, intraparietal sulcus and lateral prefrontal cortex including inferior frontal gyrus (IFG) were activated during both tasks (Fig. 2). Finally, univariate analyses did not reveal main effects of task or main effect of place of articulation.

### Functional MRI decoding results

To investigate the cortical representation of place of articulation features during the two attentional conditions (i.e., attend to consonants and attend to vowels), we implemented a classification method that relied on generalizing the discriminability of two places of articulation features (i.e., bilabial/labiodental and alveolar) across variation of three manner of articulation features (i.e., stop, fricative, and nasal).

**Figure 3.**
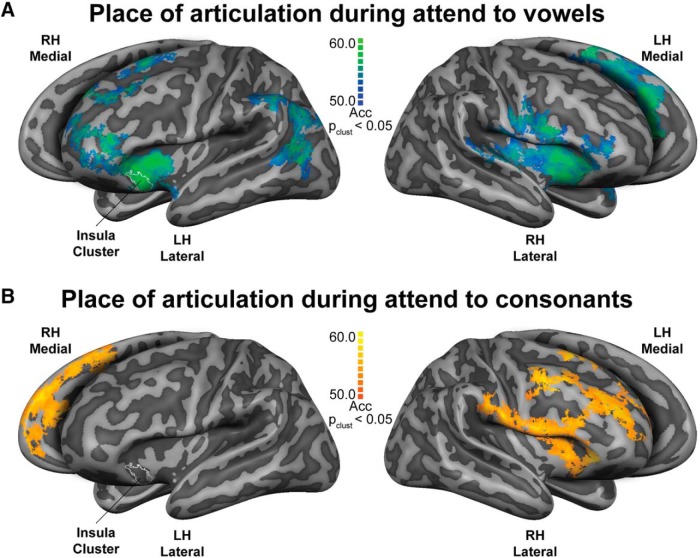
Cortical representation of place of articulation features, generalized across manner of articulation. Generalization maps depicting classification accuracies during (***A***) attend to vowels and (***B***) attend to consonants tasks. Insula cluster represents the outline (i.e., while line) of the largest continuous uncorrected cluster from the differences between the two tasks; *p* value at cluster-size threshold (p_clust_) < 0.05. Searchlight radius 8.25 mm. Maps are visualized on inflated and aligned group-averaged representations of the left (LH) and right (RH) hemispheres of the fourteen subjects (light gray, gyri; dark gray, sulci). Color scale indicates classification accuracy (Acc) percentages.

Generalization maps after correction for multiple comparisons using cluster-size thresholding (*p*-clust < 0.05) during the performance of the attend to vowels task are presented in Figure 3*A*. The generalization maps revealed successful decoding of place of articulation within regions of the brain’s language network, bilaterally. In the left hemisphere, clusters were distributed across different regions including posterior temporal, temporo-parietal, insular, anterior infero-frontal, frontal, and premotor medial regions. Specific regions included the posterior STS, supramarginal gyrus (SMG), temporoparietal junction, supplementary motor area (SMA), anterior insula, and anterior portion of the IFG (aIFG; pars orbitalis and pars triangularis). In the right hemisphere, clusters included superior temporal, insular, inferior-motor and inferior-frontal regions. Specific regions encompassed mid-posterior superior temporal plane (mSTG and pSTG), including HG, inferior central sulcus, subcentral gyrus, anterior insula, and posterior (pars opercularis) and anterior (pars triangularis and orbitalis) parts of the IFG.

Generalization maps for place of articulation during the performance of the attend to consonants task revealed significant clusters in motor regions, insula, and anterior frontal regions in the right hemisphere only (Fig. 3*B*). More specifically, these clusters included the anterior-superior portion of the angular gyrus (AG), inferior precentral gyrus, inferior central sulcus, subcentral gyrus, anterior-superior insula, posterior IFG, superior frontal sulcus, and anterior middle frontal gyrus.

To test for the modulation of place of articulation feature representations between tasks, we compared the classification accuracies of these representations within each searchlight across the two tasks using a two-sample permutation test. This analysis yielded no significant clusters with correction for cluster-size in any of the hemispheres.

**Figure 4.**
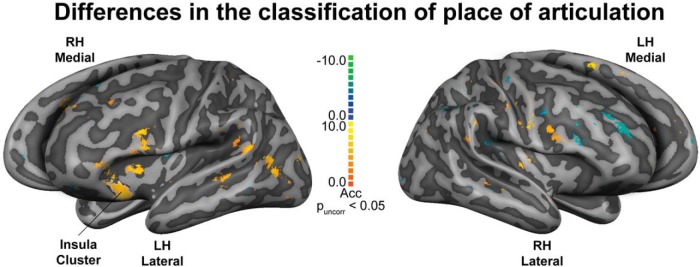
Differences in classification of place of articulation features, generalized across manner of articulation. Maps depicting the difference in the classification accuracies between attend to vowels and attend to consonants tasks. The largest continuous cluster (i.e., area, 134 mm^2^) was found in the left anterior insula, outlined in white; *p* value uncorrected (p_uncorr_) < 0.05. Searchlight radius 8.25 mm. Maps are visualized on inflated and aligned group-averaged representations of the left (LH) and right (RH) hemispheres of the fourteen subjects (light gray, gyri; dark gray, sulci). Yellow-orange clusters show larger classification accuracies during the attend to vowels task, and green-blue clusters show lager classification accuracies during attend to consonants task. Please note that the color scale does not directly relates to the colors used in Figure 3. Color scale indicates differences in the classification accuracy (Acc) percentages.

To explore possible tendencies in the data beyond the rigorous cluster-size thresholding correction, we examined the maps without correction (i.e., *p* < 0.05 uncorrected). We observed that the left hemisphere had more information (clusters) than the right hemisphere. Particularly, we found a large continuous cluster of 134 mm² located in the left anterior insula. For this cluster, the classification of place of articulation features during the attend to vowels task exhibited higher accuracies than the classification of place of articulation features during the attend to consonants task (Figs. 3, 4, white outline). Aditionally, the posterior IFG bilaterally tended to show larger classification during attend to vowels than during attend to consonants task (Fig. 4).

**Figure 5.**
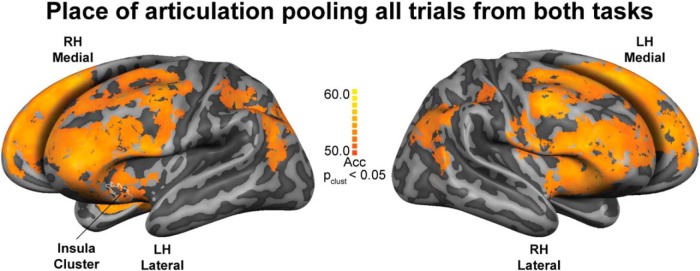
Cortical representation of place of articulation features, generalized across manner of articulation, pooling all trails from two tasks. Generalization maps depicting classification accuracies calculated by pooling all trails from attend to vowels and attend to consonants tasks. Corrected for multiple comparison with cluster-size thresholding. Insula cluster represents the outline (i.e., white line) of the largest continuous uncorrected cluster from the differences between the two tasks; *p* value at cluster-size threshold (p_clust_) < 0.05. Searchlight radius 8.25 mm. Maps are visualized on inflated and aligned group-averaged representations of the left (LH) and right (RH) hemispheres of the fourteen subjects (light gray, gyri; dark gray, sulci). Color scale indicates classification accuracy (Acc) percentages.

To increase the power in decoding at the single subject level, we renounced to task specificity by decoding the representation of place of articulation independently of the task effects. We pooled together all the trials from the attend to vowels and attend to consonants tasks and performed the classification of place of articulation ignoring whether a trial belonged to the attend to vowels or attend to consonants task. Using twice as many data as in the task-specific analysis, we expected this classification to be more robust and hence yield a higher number of significant clusters. We found bilateral clusters in the angular, supramarginal, and inferior frontal (i.e., pars opercularis and triangularis) gyri, the anterior insula, middle frontal rostral areas, middle portion of the superior frontal and middle frontocaudal areas, and the intraparietal sulcus. We also found clusters in the right posterior portion of the MTG, the left precentral gyrus, and the right subcentral gyrus (Fig. 5).

## Discussion

The present study investigated the spatial representation of place of articulation features during the attentive perception of spoken CV syllables (i.e., attend to vowels and attend to consonants). Using identical acoustic input and generalization across variation due to manner of articulation, we provided evidence for cortical representation of place of articulation features. The generalization across manner of articulation allowed us to maximize the acoustic invariance in fMRI classification (Correia et al., 2015) to counteract the differences in acoustic properties (e.g., the variance of the second formant frequency of the vowel segment due to perseverative coarticulation) that often accompany those in place of articulation (Ladefoged and Johnson, 2010).We found representation of place of articulation in separate tasks as well as in the pooled set (i.e., with both attention tasks). During the attend to vowels task, generalization maps of place of articulation features indicated feature sensitivity within bilateral clusters in superior and posterior temporal, insular, and prefrontal regions. During the attend to consonants task, place of articulation features was represented in temporoparietal, insular, and frontal regions within the right hemisphere only. The representation of place of articulation features independent of task effects (i.e., performing the generalization analysis in the pooled data) showed similar clusters to those obtained with each task separately. In addition, this analysis yielded bilateral effects as well as a more prominent contribution of frontal regions.

We observed that the brain represents the different features of place of articulation (i.e., bilabial/labiodental and alveolar) of speech sounds during attentive listening, as shown by generalization across manner of articulation. This observation supports the relevance for place in the context of sensorimotor representation of speech perception (Pulvermuller et al., 2006; Correia et al., 2015). The present result further supports previous findings that report within-place feature differentiations in STS without generalization across manner (Davis and Johnsrude, 2003; Scott and Johnsrude, 2003; Eulitz and Lahiri, 2004).

From a descriptive point of view, the cortical representation included temporal and frontal regions during the attend to vowels task, and right middle and inferior frontal regions during the attend to consonants task. This observation can be discussed in the light of differences in (1) phonological feature specification for vowels and consonants, (2) stimulus processing for consonants and vowels over time, and (3) attentional processing across the stimuli. Consonants belonged to a *specific* feature class (i.e., bilabial/labidental or alveolar), whereas vowels were equally represented across *all* syllables and place of articulation classes (Fig. 1*A*; Eulitz and Lahiri, 2004; Scharinger et al., 2016). The temporal processing of consonants and vowels in a CV structure could also have influenced the spatial distribution of the cortical representation; it has been long recognized that the syllable onset has the most relevant status in speech comprehension, whereas stimulus endings can be ignored more easily (Marslen-Wilson and Zwitserlood, 1989).

The observed cortical representation could also have related to attentional processing and variation of relevance-based selection across the two tasks. Attending to vowels meant expecting the target to occur always during the last part of the syllable. However, although the information related to the consonants is irrelevant for the task, preceding (irrelevant) linguistic information cannot be ignored, as has been shown by studies on subliminal priming (Schütz et al., 2007) and phonological priming (Bles and Jansma, 2008). Therefore, consonant-related information could have still been linguistically processed during attention to vowels, thereby impacting the respective representations within the language system. Attending to consonants meant, in turn, expecting the target to occur always during the first part of the syllable, so the information presented after the target (i.e., the vowel) could be easily ignored or discarded. Consequently, the cortical representation of place of articulation features during the attend to vowels task was found to include all areas initially expected for the attend to consonants task (e.g., Correia et al., 2015). Similarly, consonant to vowel transition features may have been amplified during the attend to vowels task, involving specific bilateral temporal regions (Humphries et al., 2014). As during the attend to consonants task no transition (to a subsequent vowel) should be amplified, brain regions dealing with such transitions were not engaged. Future studies could also include other sequences of syllable components (e.g., vowel-consonant syllables or CV-consonant structures) to clarify the effect of the relevance-based selection.

An alternative possibility pertains to general-feature sensitivity of the language system during attention to vowels and specific-feature selectivity during attention to consonants, as both syllable components can exhibit acoustic and articulatory properties (Ladefoged and Johnson, 2010). Given their relatively clearer acoustic features (Narayan et al., 2010), vowels could have had relatively higher perceptual (i.e., auditory) saliency in comparison with consonants, thus explaining the extended representation of place of articulation features during the attention to vowels task. However, this possibility is unlikely, because our participants did not differ in reaction times between tasks, and higher saliency should result in faster reaction times during detection and match to sample tasks.

The comparison between the two classification maps of place of articulation features yielded a large continuous cluster (uncorrected) in the left anterior insula for attention to vowels versus attention to consonants. This result points toward a role of the anterior insula in the modulation of the representation of place of articulation, determined by selective attention to vowels in CV syllables. A relevance-based selection, resulting from the specificity of our design, might have dictated a task set where attention to vowels required relatively greater demands on top-down selectivity (i.e., in the attempt to ignore the first part of the syllable). This interpretation is in agreement with the role of the anterior insula as part of a task-set system (Dosenbach et al., 2006) in volitional top-down control (Nelson et al., 2010) and alertness (Sadaghiani et al., 2010; Clemens et al., 2011). Moreover, the anterior insula has been incorporated within the hierarchy of the language network (Davis and Johnsrude, 2003), for example with a role in articulatory planning (Dronkers, 1996; Baldo et al., 2011).

The lack of significant clusters representing place of articulation features in other parts of the temporal lobe (e.g., primary auditory cortex) was expected given our analysis approach. The main purpose of combining MVPA with generalization was to maximize the extraction of the information about the abstract (higher order) features under study (i.e., place of articulation) while minimizing the impact of variation in acoustic (i.e., manner of articulation) information. Thus, the clusters show where information about place of articulation is represented, free from perceptual properties and information processing of the sounds. Moreover, the univariate analysis results support a critical role of the auditory cortices in speech perception and auditory processing (Fig. 2). However, it should be noted that the univariate maps are showing the main effect to sound compared to silence (i.e., overall relative changes of activation in cortical regions in response to sound). Therefore, these maps reflect sound responsive areas (to speech in our experiment), rather than isolated language-specific areas. The representation of place of articulation revealed by the generalization analysis in separate tasks was also confirmed when the trials from the two tasks were pooled. The additional clusters in frontal areas could have resulted from both the increase in the statistical power (i.e., using twice the number of trials in comparison to the task-specific analyses) and the higher sensitivity of multivariate pattern analysis (i.e, compared to univariate analysis; Kriegeskorte et al., 2006; Norman et al., 2006; Mur et al., 2008).

Overall, our generalization approach showed a network of distributed brain regions (including frontal and sensorimotor areas), where articulatory features are represented during the perception, attention, and short-term memory storage within the delay-match-to-sample tasks. This finding fits well with the cortical areas critical for language comprehension (e.g., the angular and supramarginal gyri; Turken and Dronkers, 2011), sensorimotor integration (Pulvermuller et al., 2006; Hickok et al., 2011; Schomers and Pulvermüller, 2016), and semantic knowledge retrieval (e.g., AG; Dronkers et al., 2004; Binder et al., 2009). Moreover, lesion studies showing sentence comprehension impairment have also suggested a role for dorsolateral frontal areas in auditory information integration [e.g., short-term verbal memory rehearsal during sentence comprehension (Smith and Jonides, 1999) and the representation of speech sequences (Fuster, 2001)].

Considering the observed representation of place of articulation, it would seem reasonable to analyze the individual phonological features in an independent manner. However, we could not do so within our dataset due to the setup of the current experiment, in which the total number of trials was limited. Limitations were related to the sparse sampling acquisition necessary to avoid scanner noise interference, which increased the trial duration. In addition, stimuli tokens were reduced to a subset required to introduce enough variance for classification. These choices, though necessary for the purpose of the study, resulted in selection of material with few individual place of articulation features. Previous studies have also shown representation of individual abstract phonological features (Obleser et al., 2003, 2004), notions of underspecification (Scharinger et al., 2016), as well as a multidimensional space of features (i.e., spectral peak or voice onset time) in the encoding of acoustic parameters of speech (Mesgarani et al., 2008, 2014).

In summary, here we showed classification of place of articulation features generalized across manner of articulation for the first time. Our results provide evidence for cortical representation of place of articulation features during attentive syllable perception (i.e., attention to the different syllable components) in sensorimotor, temporoparietal, and frontal areas within the language network. This cortical representation was revealed in additional clusters (e.g., bilateral frontal and sensorimotor areas) when increasing the statistical power. Additionally, we observed a more bilateral distribution for attend to vowels, a more unilateral distribution for attend to consonants, and a trend toward a modulatory role of the left anterior insula in selective attention of speech sounds. To conclude, these data support sensorimotor integration during attentive speech perception and demonstrate that a generalization approach can be used to exclude “common factors,” such as perceptual properties, from the analysis.

## Synthesis

Reviewing Editor: Morgan Barense, University of Toronto Decisions are customarily a result of the Reviewing Editor and the peer reviewers coming together and discussing their recommendations until a consensus is reached. When revisions are invited, a fact-based synthesis statement explaining their decision and outlining what is needed to prepare a revision will be listed below. The following reviewer(s) agreed to reveal their identity: Bradley Buchsbaum.

We agreed that the authors have done a good job addressing previous reviews. However, there are a few remaining points which the authors should consider:

1. The attend-vowel attend-consonant maps appear, superficially at least, to be quite different. However, a direct contrast reveals no differences, at least when (full) multiple comparisons correction is used. This may mean that the very different looking maps are misleading and the result of small differences that are magnified by the thresholding operation. For this reason it would be nice to see the main effect map -- the map based on pooling over attend-consonant attend-vowel. If there is no real underlying interaction, this map will be better because it is based on more samples. Another approach is to use a conjunction analyses to show areas that generalize over attention.

2. Related to the above. In the attend vowel contrast, we see ‘blue’ areas that are outside the language area, at least as defined by the univariate contrast. For example, in the angular gyrus and superior frontal cortex. Does this even make sense? Are there regions representing abstract phonological features that don't ‘activate’ during speech? Moreover, do lesions to the e.g. angular gyrus--outside the auditory responsive zone-- cause speech perception deficits that selectively affect place of articulation? If not, would the authors make the prediction, based on the present data, that TMS to the superior frontal gyrus would affect speech perception of place of articulation?
